# FitSNPs: highly differentially expressed genes are more likely to have variants associated with disease

**DOI:** 10.1186/gb-2008-9-12-r170

**Published:** 2008-12-05

**Authors:** Rong Chen, Alex A Morgan, Joel Dudley, Tarangini Deshpande, Li Li, Keiichi Kodama, Annie P Chiang, Atul J Butte

**Affiliations:** 1 Stanford Center for Biomedical Informatics Research, 251 Cmpus Drive, Stanford, CA 94305, USA; 2 Department of Pediatrics, Stanford University School of Medicine, Stanford, CA 94305, USA; 3 Lucile Packard Children's Hospital, 725 Welch Road, Palo Alto, CA 94304, USA; 4 NuMedii Inc., Menlo Park, CA 94025, USA

## Abstract

**Background:**

Candidate single nucleotide polymorphisms (SNPs) from genome-wide association studies (GWASs) were often selected for validation based on their functional annotation, which was inadequate and biased. We propose to use the more than 200,000 microarray studies in the Gene Expression Omnibus to systematically prioritize candidate SNPs from GWASs.

**Results:**

We analyzed all human microarray studies from the Gene Expression Omnibus, and calculated the observed frequency of differential expression, which we called differential expression ratio, for every human gene. Analysis conducted in a comprehensive list of curated disease genes revealed a positive association between differential expression ratio values and the likelihood of harboring disease-associated variants. By considering highly differentially expressed genes, we were able to rediscover disease genes with 79% specificity and 37% sensitivity. We successfully distinguished true disease genes from false positives in multiple GWASs for multiple diseases. We then derived a list of functionally interpolating SNPs (fitSNPs) to analyze the top seven loci of Wellcome Trust Case Control Consortium type 1 diabetes mellitus GWASs, rediscovered all type 1 diabetes mellitus genes, and predicted a novel gene (*KIAA1109*) for an unexplained locus 4q27. We suggest that fitSNPs would work equally well for both Mendelian and complex diseases (being more effective for cancer) and proposed candidate genes to sequence for their association with 597 syndromes with unknown molecular basis.

**Conclusions:**

Our study demonstrates that highly differentially expressed genes are more likely to harbor disease-associated DNA variants. FitSNPs can serve as an effective tool to systematically prioritize candidate SNPs from GWASs.

## Abstract 2

Differential expressed genes are more likely to have variants associated with disease. A new tool, fitSNP, prioritizes candidate SNPs from association studies.

## Background

A major goal of biomedical research is to identify genes that contribute to the molecular pathology of specific diseases. This process has been accelerated by two types of high-throughput studies: genome-wide association studies (GWASs) and gene expression microarray studies. A GWAS scans a genome for single nucleotide polymorphisms (SNPs) associated with disease, whereas microarrays identify genes that are differentially expressed between disease and control samples. These methods have been integrated into molecular profiling to identify expression quantitative trait loci and to build pathways that are involved in various diseases, including type 2 diabetes [1,2], atherosclerosis [3], dystrophic cardiac calcification [4], metabolic disorders [5], and cardiovascular disorders [6]. To lower the cost, GWASs are frequently designed as a two-stage study [7]; first is a stage involving identification of candidate SNPs, and then a validation stage is conducted, in which the effect of the candidate SNPs in a larger population is determined. However, in a recent two-stage GWAS of prostate cancer, most of the SNPs determined to be significant were not even ranked in the top 1,000 SNPs in the identification stage [7], which suggests that existing candidate SNP prioritization methods, which are largely based on known functional annotations, are inadequate.

There are many candidate gene and SNP prioritization methods, including the use of sequence information [8,9], protein-protein interaction networks [10,11], literature and ontology [12,13], and various combination of these methods [14]. For a detailed description of the available tools, the reader is referred to comprehensive reviews [15,16]. Gene expression is often taken into consideration when prioritizing candidate genes or SNPs, but this is most often within the context of the specific disease, such as disease-related anatomical regions and tissue specificity [17-20], conserved co-expression [21], coherent expression profile with known disease-associated genes [22], or several expression datasets in model organisms [23]. These disease-specific gene expression prioritization methods are somewhat informative, but they are cumbersome, requiring extensive manual work. Given that there are more than 200,000 microarray studies included in the National Center for Biotechnology Information's Gene Expression Omnibus (GEO) [24] and more than 10,000 disease-associated DNA variants in the Genetic Association Database (GAD) [25] and Human Gene Mutation Database (HGMD) [26], we hypothesize that a more general (and therefore more systematic) link exists between a gene's expression and the likelihood that it is associated with disease.

Recognizing the wealth of gene expression data in public repositories, we propose an integrative genomics method to systematically prioritize DNA markers that aims to accelerate the identification of novel causative genes and variants. Here, we analyzed every available human microarray study in GEO; we calculated the frequency of differential expression for every gene; and we found that the more often a gene was differentially expressed, the more likely it was that it contained disease-associated variants. Based on this discovery, we derived a list of functionally interpolating SNPs (fitSNPs) from differential gene expression, and we showed how fitSNPs could have been used to successfully prioritize genes from type 1 and type 2 diabetes mellitus GWASs, as well as previously identified Online Mendelian Inheritance in Man (OMIM) loci with unknown molecular basis.

## Results

### Highly differentially expressed genes are more likely to harbor disease-associated variants

In order to determine whether differentially expressed genes are genetically associated with disease, we downloaded all 476 curated human GEO datasets to serve as our human gene expression set. The probes from these GEO datasets, which include groups of microarrays organized by experimental variable (for example, time, tissue, agent, temperature, and so on), were annotated with the latest National Center for Biotechnology Information Entrez Gene annotations using AILUN [27]. We conducted 4,877 group-versus-group comparisons using significance analysis of microarrays (SAM) [28] and obtained a list of 19,879 genes that were differentially expressed with *q *value under 0.05 in one or more experiments. We then created a list of curated human disease-associated genes by combining GAD [25] and HGMD [26], resulting in a list of 3,221 genes with disease-associated variants.

We compared our list of differentially expressed genes with the list of genes with disease-associated variants, and we found that 99% of disease-associated genes were differentially expressed in one or more GEO datasets, with 14% specificity (Additional data file 1). The likelihood of having variants associated with disease was 12 times higher among differentially expressed genes than among constantly expressed genes (*P *< 0.0001, Fisher's exact test), whereas the likelihood of having a nonsynonymous coding SNP was 1.6 times higher among differentially expressed genes than among constantly expressed genes.

In order to characterize better the relationship between DNA variance and expression in all human genes, we tested whether genes differentially expressed in multiple microarray studies are more likely to have disease-associated variants. For each gene, a differential expression ratio (DER) was calculated as the count of GEO datasets in which it was differentially expressed (*q *value ≤ 0.05) divided by the count of GEO datasets in which it was measured. The calculation was restricted to genes that were measured in at least 5% of all GEO datasets.

**Figure 1:**
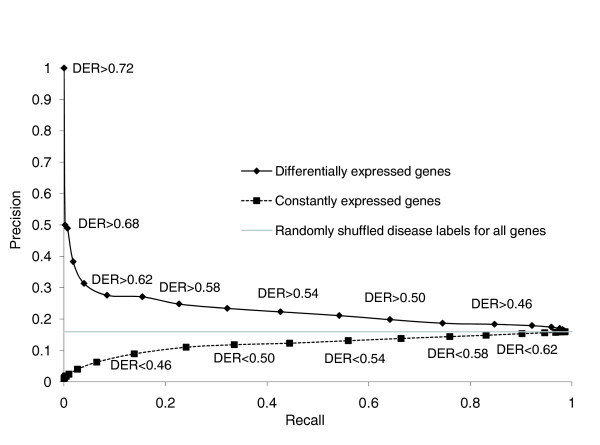
Use of differentially and constantly expressed genes to rediscover disease genes. The DER was calculated as the count of GEO datasets in which a gene was differentially expressed divided by the count of GEO datasets in which it was measured. For any cutoff *x*, differentially expressed genes were defined as genes with DER > *x*, whereas constantly expressed genes were defined as genes with DER <*x*. The precision/recall graphs show that the likelihood of harboring disease mutations for a gene increases when its DER value increases. For the control, we shuffled disease labels 10,000 times among all genes and obtained a predicted precision of 16%. DER, differential expression ratio; GEO, Gene Expression Omnibus.

The precision of rediscovering a disease gene was 16% for genes with a DER greater than 0. This precision improved gradually to 28% when the DER was greater than 0.62, and then increased dramatically to 100% when the DER was greater than 0.72 (Figure 1). As a control, a similar graph is also plotted in Figure 1 for constantly expressed genes with a DER less than the cutoffs used. The more GEO datasets in which a gene was constantly expressed, the less likely it was to contain disease-associated variants. As an additional control, we randomly shuffled disease labels for all genes 10,000 times, and the precision of rediscovering disease genes remained at the predicted 16%. Compared with constantly expressed or randomly shuffled disease genes, the more often a gene was differentially expressed, the more likely it was that it contained DNA variants associated with diseases.

**Figure 2:**
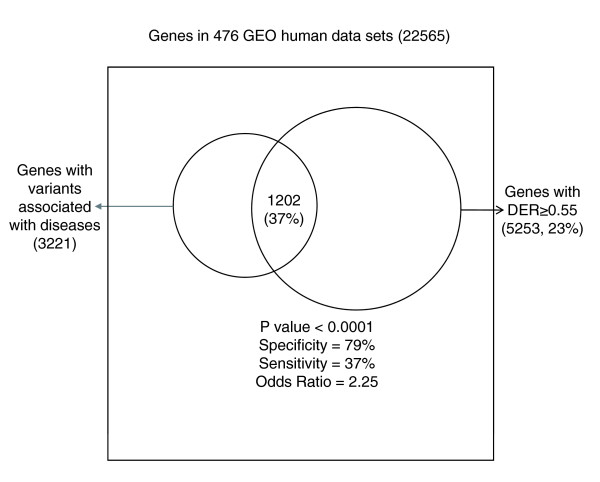
Performance of rediscovering disease genes by DER. Genes with DER ≥ 0.55 were predicted to be disease genes, and compared with genes with disease-associated DNA variants listed in GAD and HGMD. *P *values were calculated using Fisher's exact test. DER, differential expression ratio; GAD, Genetic Association Database; GEO, Gene Expression Omnibus; HGMD, Human Gene Mutation Database.

In a receiver operating characteristic curve constructed to rediscover disease genes using the DER values, a DER value ≥ 0.55 exhibited the best performance, with 79% specificity and 37% sensitivity. As shown in Figure 2, genes with DER ≥ 0.55 were 2.25 times more likely to harbor disease-associated variants than others (*P *< 0.0001, Fisher's exact test). Varying the threshold, we achieved 56% specificity and 65% sensitivity at DER ≥ 0.50, and 93% specificity and 16% sensitivity at DER ≥ 0.60.

### DER distinguishes true type 1 diabetes mellitus genes from false positive genes in GWASs

The likelihood of harboring disease-associated variants in genes with high DER values could be used to prioritize candidate SNPs from GWASs. To lower the cost, GWASs are often designed as a two-stage experiment: identifying candidate SNPs and then validating them in a larger population. Most often, functionally important genes are manually selected from the loci around positive SNPs for sequencing or high-quality genotyping in a larger population. This prior knowledge based gene prioritization method is not only time consuming but is also likely to miss novel genes. Indeed, associations for a large number of candidate genes from identification stage of GWASs were found to be false positives in the validation stage. A test to distinguish true disease genes from these false-positive genes will demonstrate the prioritizing power of DER in GWASs.

**Figure 3:**
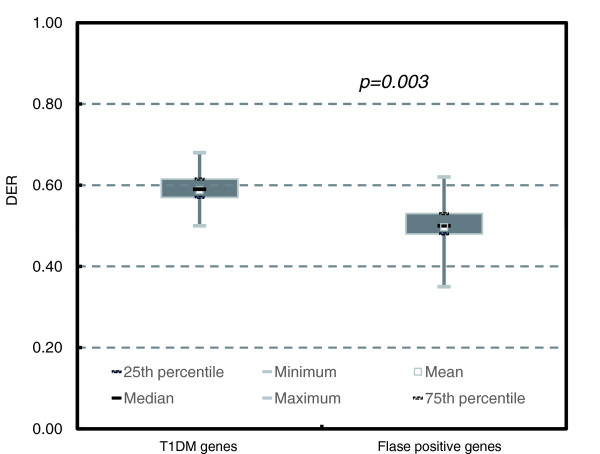
Distinguishing T1DM genes from false positives in the top seven loci from GWASs using DER. Genes in the top seven loci from the WTCCC T1DM GWASs are reported with validation results. False-positive genes were shown as positive in the initial scan but found to be unassociated with T1DM in the follow-up validation studies. T1DM genes had significantly higher DER values than did false positive genes (*P *= 0.003). The mean DER values for T1DM and false-positive genes were 0.59 and 0.50, respectively. DER, differential expression ratio; GWAS, genome-wide association study; T1DM, type 1 diabetes mellitus; WTCCC, Wellcome Trust Case Control Consortium.

We first evaluated the performance in type 1 diabetes mellitus (T1DM). Within the top seven T1DM loci (6p21, 12q24, 12q13, 16p13, 18p11, 12p13, and 4q27) identified from the Wellcome Trust Case Control Consortium (WTCCC) GWAS [29], 21 genes were reported with genotyping results in two follow-up studies [30,31]. Table 1 lists their DER values along with their validation results. As shown in Figure 3, the DER values of T1DM genes were significantly higher than those for false-positive genes (*P *= 0.003, *t*-test), with clear separation of the 25th to 75th percentile ranges. Among the ten genotyped candidate genes with DER ≥ 0.55, all but *ITPR3 *were validated as true T1DM genes. Of the 11 genotyped genes with DER < 0.55, all but three (*HLA-DPB1*, *C12orf30*, and *KIAA0350*) were found to be unassociated with T1DM. We successfully distinguished true T1DM genes from false positives with 89% specificity and 75% sensitivity (*P *= 0.02, Fisher's exact test). If we only genotype genes with DER ≥ 0.50, then we identify all true T1DM genes, with a 56% false discovery rate.

**Table 1:** DER values for T1DM and false positive genes in the top 7 WTCCC T1DM loci

Loci	Gene^a^	Associated?^b^	DER	Correct?^c^
4q27	*TENR*	No	0.54	True negative

4q27	*IL2*	No	0.48	True negative

4q27	*IL21*	No	0.46	True negative

6p21	*HLA-DQB1*	Yes	0.68	True positive

6p21	*HLA-DRB1*	Yes	0.61	True positive

6p21	*HLA-B*	Yes	0.59	True positive

6p21	*HLA-A*	Yes	0.59	True positive

6p21	*HLA-DPB1*	Yes	0.54	False negative

6p21	*TAP2*	Yes	0.58	True positive

6p21	*CFB*	Yes	0.59	True positive

6p21	*MICA*	No	0.5	True negative

6p21	*MICB*	No	0.53	True negative

6p21	*MASIL*	No	0.35	True negative

6p21	*UBD*	No	0.48	True negative

6p21	*ITPR3*	No	0.62	False positive

12p13	*CLEC2D*	No	0.51	True negative

12q13	*ERBB3*	Yes	0.63	True positive

12q24	*C12orf30*	Yes	0.52	False negative

12q24	*SH2B3*	Yes	0.58	True positive

16p13	*KIAA0350*	Yes	0.5	False negative

18p11	*PTPN2*	Yes	0.64	True positive

^a^The positive candidate genes from WTCCC GWAS with reported validation results. ^b^Validated to be associated or unassociated with T2DM in the high-quality genotyping. ^c^The predicted result using DER ≥ 0.55. DER, differential expression ratio; GWAS, genome-wide association study; T1DM, type 1 diabetes mellitus; WTCCC, Wellcome Trust Case Control Consortium.

### DER distinguishes true type 2 diabetes mellitus genes from false-positive genes in GWASss

To validate the robustness of this method, we applied it to another disease, namely type 2 diabetes mellitus (T2DM), which had been studied in six large-scale GWASs [29,32-36] and tens of targeted association studies in more than 20 populations. We extracted all significant T2DM genes described in the abstracts, and limited the list to those with significant association in at least three different populations, and derived 15 widely accepted T2DM genes (Table 2). We also retrieved SNPs that were reported to exhibit significant association in the identification stage but no association in the validation stage in a large-scale T2DM GWAS [32]. We annotated these negative SNPs with their associated genes using Entrez dbSNP, and we removed those without gene annotations, and derived 13 negative genes. As shown in Table 2, DER ≥ 0.55 successfully distinguished T2DM genes from negative genes with 85% specificity and 60% sensitivity (*P *= 0.02, Fisher's exact test).

**Table 2:** DER values for T2DM and false positive genes from GWAS

Locus or SNP	Gene	Associated in populations	DER	Correct?^a^
2q37.3	*CAPN10*	Finish. [55], Korean [56], Mexican. [55], Tunisian [57]	0.57	True positive

3p25	*PPARG*	Caucasian. [58], Finish. [59], German. [60], Indian Sikhs. [61], Japanese. [62], Mexican. [63]	0.53	False negative

3q27.2	*IGF2BP2*	Asian. [64], Caucasian. [33], Chinese [65], Danish. [66], French. [67], German. [60], Hispanic. [68], Indian Sikhs. [61], Japanese. [69], Norwegian. [70]	0.54	False negative

6p22.3	*CDKAL1*	Asian. [64], Ashkenazi Jewish. [71], Caucasian. [33], Chinese [65], German. [60], Hispanic. [68], Japanese. [69], Norwegian. [70]	0.55	True positive

8q24.11	*SLC30A8*	Asian. [64], African. [68], Caucasian. [33], Chinese [65], Hispanic. [68], Japanese. [69], Norwegian. [70]	0.42	False negative

9p21	*CDKN2A*	Asian. [64], Caucasian. [34], Chinese [65], Danish. [66], French [72], Japanese. [69]	0.59	True positive

9p21	*CDKN2B*	Asian. [64], Caucasian. [33], Chinese [65], Danish. [66], French [72], Japanese. [69], Norwegian. [70]	0.49	False negative

10q23	*HHEX*	Asian. [64], Caucasian. [33], Chinese [65], Danish. [66], German. [60], Japanese. [69], Norwegian. [70]	0.58	True positive

10q23	*IDE*	Caucasian. [73], Chinese [65], Danish. [66], Japanese. [74], Korean. [75]	0.61	True positive

10q24	*KIF11*	Caucasian, Chinese [65], Danish. [66], Japanese. [74]	0.54	False negative

10q25.3	*TCF7L2*	African. [76], Ashkenazi Jewish. [71], Asian. [64], Caucasian. [33], Chinese. [77], German. [60], Hispanic. [78], Indian Sikhs. [61], Japanese. [79], Spanish, UK white. [80]	0.64	True positive

11p15.1	*KCNJ11*	Arab. [81], Caucasian. [33], Czech [82], Japanese. [69]	0.39	False negative

11p15.5	*KCNQ1*	Singaporean. [35], European. [35], Japanese. [35]	0.6	True positive

16q12.2	*FTO*	Asian. [64], Caucasian. [34], Indian Sikhs. [61], German. [60], Japanese. [83], Norwegian. [70]	0.55	True positive

20q12	*HNF4A*	Amish. [84], Ashkenazim [85], Danish. [86], Finish. [87], Swedish. [87], Mexican. [88], Norwegian. [89], UK Caucasian. [90]	0.63	True positive

rs11078674	*NLGN2*	No	0.53	True negative

rs2866016	*TSPAN5*	No	0.53	True negative

rs12629276	*RFTN1*	No	0.54	True negative

rs8101509	*ZNF649*	No	0.4	True negative

rs945384	*FAM69B*	No	0.53	True negative

rs2050831	*VPS13A*	No	0.63	False positive

rs6670163	*RYR2*	No	0.55	False positive

rs859101	*SLC44A3*	No	0.5	True negative

rs11084127	*ZNF615*	No	0.46	True negative

rs7950175	*KIRREL3*	No	0.48	True negative

rs13064991	*SLC6A20*	No	0.45	True negative

rs6541240	*TTC13*	No	0.51	True negative

rs2278419	*ZNF350*	No	0.45	True negative

^a^The predicted result using DER ≥ 0.55. DER, differential expression ratio; GWAS, genome-wide association study; T2DM, type 2 diabetes mellitus.

### FitSNPs predicts T1DM genes directly from the top seven WTCCC T1DM loci

The robustness of DER to distinguish disease genes from false positives in T1DM and T2DM GWASs led us to hypothesize that it may also be used to predict disease genes directly from the loci identified from GWASs. To facilitate the visualization of DER values along with GWAS results on the human genome, we created a tool called functionally interpolating SNPs (fitSNPs) [37]. It is a list of human SNPs with DER values assigned according to their associated genes. It can be easily loaded into the University of California Santa Cruz (UCSC) genome graph [38] and visualized on the human genome along with a wealth of preloaded or user-defined genomic data, such as GWAS results. We called the tool 'functionally interpolating SNPs' because it not only infers the likelihood of disease association for all human SNPs but also suggests potential diseases to guide functional studies. In the Gene page of the FitSNPs server, clicking the DER value for any gene will display all biologic and clinical conditions in which it was found to be differentially expressed, with statistical comparisons and filter/sort functions [39].

We therefore examined each of the top seven WTCCC T1DM loci on the UCSC genome browser to evaluate whether we could predict T1DM genes using fitSNPs. The hypothesis is that a gene with a significantly higher DER value than other genes in the vicinity will probably explain the observed disease association from the locus.

In 12q13, *ERBB3 *is the only gene with high scores in both the WTCCC T1DM GWAS and fitSNPs, and this gene was indeed found to contain rs2292239, which is the only confirmed T1DM marker within this region. In 18p11, *PTPN2 *is the only gene suggested by fitSNPs (DER = 0.64), and it was confirmed to explain the association with T1DM for this region. In 16p13, we predicted *SOCS1 *to be the most significant gene (DER = 0.64), and the follow-up study showed that it contains the validated marker rs243329 (-log_10_*P *= 4.19). However, we missed *KIAA3350 *(DER = 0.5) from 16p13, which has a confirmed association with T1DM and a higher -log_10_*P *than *SOCS1*. In 12p13, no gene has a high score in both GWAS and fitSNPs, which is consistent with the fact that no association was found in the follow-up parent-child trio study [31].

Within 12q24, *SH2B3 *and *ALDH2 *have high scores in both T1DM and fitSNPs, and indeed *SH2B3 *was confirmed to contain a mutation in R262W that explains the association with T1DM in this region in the follow-up study [31]. The association of *SH2B3 *with T1DM is somewhat fortuitous because it was originally excluded based on data quality. Only upon recovering additional, poorly clustered nonsynonymous SNPs was it screened for association. This highlights an inadequate prioritization approach, which currently is based on existing functional annotations. This gene prioritization problem is addressed by fitSNPs because it is not biased by existing functional annotations. It is not clear whether there was any follow-up study on mitochondrial aldehyde dehydrogenase 2 (the protein encoded by *ALDH2*), which detoxifies aldehydes generated by alcohol metabolism and lipid peroxidation in the mitochondrial matrix. The association of inactive *ALDH2 *genotype with maternal inheritance of T1DM, previously reported in a Japanese population [40], suggests that it may also play a role in T1DM.

**Figure 4:**
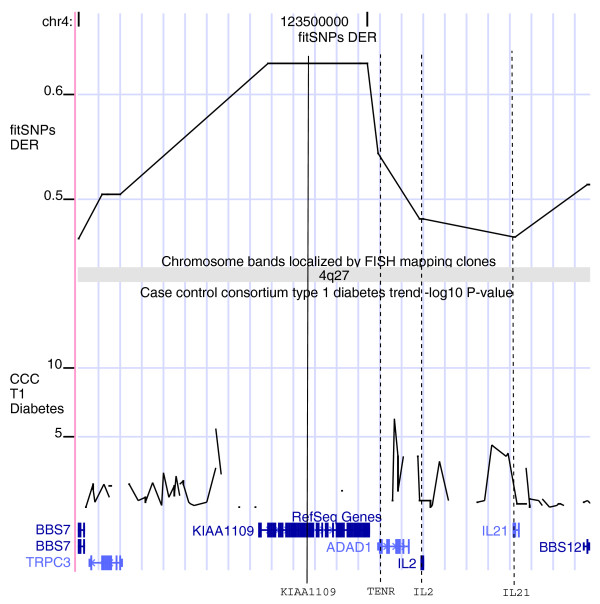
Interpreting T1DM GWAS findings at 4q27 using fitSNPs. The region 4q27 has been identified as a risk factor area for T1DM, celiac disease, and rheumatoid arthritis. *IL2*, *IL21*, and *TENR *were selected based on prior knowledge for sequencing in the follow-up studies, but no association was found. *KIAA1109 *has a much higher fitSNPs DER value than all other genes in the region, and is flanked by two significant T1DM GWAS SNPs (-log_10_*P *>5). We predicted that this gene may explain the T1DM association in this region. The GWAS -log_10_*P *curve for *KIAA1109 *is missing because it was not listed in the Affymetrix 500 K SNP array used for the GWAS. DER, differential expression ratio; fitSNPs, functionally interpolating single nucleotide polymorphisms; GWAS, genome-wide association study; SNP, single nucleotide polymorphism; T1DM, type 1 diabetes mellitus.

Within 4q27, *IL2*, *IL21*, and *TENR *were selected for deep sequencing in the T1DM follow-up study because of the association of T1DM susceptibility with *IL2 *in nonobese diabetic mice. However, no T1DM marker had been found in these three genes, and the T1DM association of 4q27 remains unexplained. Figure 4 shows the fitSNPs DER values along with T1DM GWAS -log_10_*P *at 4q27 on the UCSC genome browser [38]. We found that *KIAA1109*'s DER value (0.63) is much greater than those for all other genes in 4q27, including *IL2 *(0.48), *IL21 *(0.46), and *TENR *(0.54). It is flanked by two most significant T1DM GWAS SNPs, and is highly likely to be associated with T1DM. The -log_10_*P *curve within *KIAA1109 *was missing because it was not listed in the genotyping array used in the WTCCC T1DM GWAS (Affymetrix 500K SNP array; Affymetrix Inc., Santa Clara, CA, USA).

Interestingly, the 4q27 region has also been found to be associated with celiac disease [41] and rheumatoid arthritis [42], suggesting that it might be a general risk factor for multiple autoimmune diseases. It has been reported that rs13119723 in *KIAA109 *has the most significant association with celiac disease outside the HLA region (*P *= 2 × 10^-7^) [41]. By examining our annotated microarray database of disease versus normal gene expression datasets [43], we found that *KIAA1109 *was significantly downregulated in peripheral blood cells in juvenile rheumatoid arthritis in two independent studies [44,45]. Additionally, the GNF SymAtlas lists it as being highly expressed in T cells [46]. Therefore, *KIAA1109 *is a valuable gene for further investigation in T1DM and other autoimmune diseases, and we predict that it is likely to explain the T1DM association in 4q27.

### Comparing DER values among different types of disease genes

The success of these three validation studies demonstrates that fitSNPs could be used not only to prioritize different loci from GWASs but also to prioritize genes from each locus. Before applying fitSNPs to all diseases, one important question is whether genes associated with different type of diseases have different DER values. We downloaded lists of disease genes for Mendelian diseases (highly penetrant diseases caused by a single mutation), complex diseases, and cancer, which were compiled by Ran Blekhman and coworkers [47]. As shown in Table 3, no significant DER difference were observed between Mendelian and complex disease genes (0.53 versus 0.54; *P *= 0.2, *t*-test). Cancer genes exhibited significantly higher DER values (0.56) than did both Mendelian (*P *< 0.0001, *t*-test) and complex disease genes (*P *= 0.001, *t*-test). Furthermore, all types of disease genes exhibited significantly higher DER values than did nondisease genes (*P *< 0.0001, *t*-test). These findings suggest that fitSNPs could be used to prioritize disease genes for both Mendelian and complex diseases, and would be even more effective in prioritizing cancer genes.

**Table 3:** DER value comparisons among Mendelian, complex, cancer, all disease genes and nondisease genes

*P *value^a^	Mendelian (mean = 0.53, n = 931)	Complex (mean = 0.54, n = 70)	Cancer (mean = 0.56, n = 324)	All diseases (mean = 0.53, n = 3,178)	Nondisease (mean = 0.50, n = 16,698)
Mendelian		0.2	<0.0001	0.4	<0.0001

Complex			0.001	0.3	<0.0001

Cancer				<0.0001	<0.0001

All diseases					<0.0001

Nondisease					

*P values were calculated using *t*-test. DER, differential expression ratio.

### FitSNPs predicts disease genes in OMIM loci with unknown molecular basis

FitSNPs could be used not only to prioritize disease genes from GWASs for multiple disease types, but also to predict disease associations for genes with high DER values. There are 5,253 human genes with DER ≥ 0.55. Of these, 23% have known variants for various diseases according to GAD and HGMD. The remaining 4,052 genes have not yet been shown to associate with any diseases through mutations or polymorphisms, making them promising leads. To systematically predict disease associations for them, we searched OMIM and found that 830 diseases and syndromes have been linked to cytogenetic locations but not specific genes. From these cytogenetic locations, we predicted 3,331 highly differentially expressed genes with DER ≥ 0.55 in 610 diseases. From this group, 2,586 genes, which are currently not associated with any disease according to GAD and HGMD, were predicted to be associated with 597 diseases [48].

**Figure 5:**
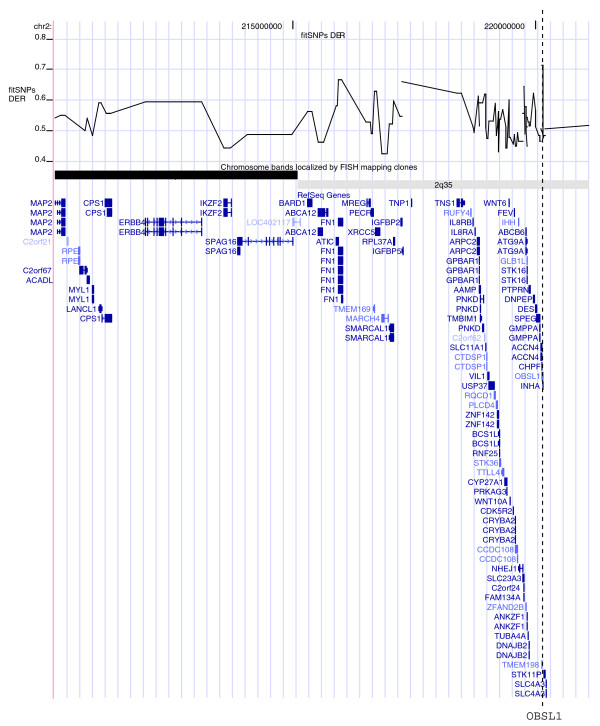
Prediction that *OBSL1 *is associated with systemic lupus erythematosus with nephritis through 2q34-q35. Systemic lupus erythemetosus with nephritis (*SLEN2*; OMIM %607966) was identified to be associated with 2q34-q35 but without identification of specific genes. *OBSL1 *has a much higher DER value (0.71) than those of all other genes from 2q34-q35. It was also found to be differentially expressed in juvenile idiopathic arthritis, kidney cancer, and kidney transplant rejection. Therefore, we suggest that it should be sequenced for its potential association with *SLEN2*.

For example, systemic lupus erythemetosus (SLE) is an autoimmune disease with multiple organ involvement and a genetic predisposition. Renal disease occurs in 40% to 75% of SLE patients and up to 90% of childhood SLE patients, and significantly contributes to morbidity and mortality. A genome scan was performed with more than 300 microsatellite markers in the 75 pedigrees that had SLE with nephritis, and linkage was identified at 2q34-q35 with *P *= 0.000001 (*SLEN2*; OMIM %607966). To date, no gene in 2q34-q35 has been associated with *SLEN2*. The DER for the gene *OBSL1 *(obscurin-like 1; DER = 0.71) is significantly greater than that for all other genes (Figure 5). Actually, it has the second highest DER value among all human genes without known disease-associated variants. By examining our annotated microarray database of disease versus normal gene expression datasets [43], we found that *OBSL1 *was significantly differentially expressed in juvenile idiopathic arthritis (GEO series 8650) and several kidney diseases, such as kidney cancer (GEO dataset 9) and kidney transplant rejection (GEO dataset 724). Therefore, we suggest that *OBSL1 *might be associated with *SLEN2*. Similarly, we suggest that the 2,586 genes predicted with DER values are top candidate genes for the 597 syndromes in question.

## Discussion

We analyzed 476 human GEO datasets and calculated the frequency of differential expression for every gene, which we called the differential expression ratio (DER). The enrichment analysis on a comprehensive list of curated disease genes revealed a positive association between DER values and the likelihood of harboring disease-associated mutations. We were able to rediscover all disease genes with 79% specificity and 37% sensitivity using a simple threshold of DER ≥ 0.55. These highly differentially expressed genes were 2.25 times more likely to harbor disease-associated variants than others. The positive association between DER and our precision to rediscover disease genes was consistently observed across ranges of DER values, in spite of variable adjustments, including adjusting the *q *value cutoff from 0.005 to 0.2, and the removal of genes measured in fewer than 0% to 30% microarray studies. Additionally, we analyzed disease genes from three different human genetic association databases, namely GAD, HGMD, and OMIM, individually and observed the same DER-related increase in precision. We also used the absolute GEO dataset counts instead of the DER to rediscover disease genes and observed the same pattern. The majority of 476 GEO datasets are genome-wide experiments; 98% of GEO datasets contained more than 5,000 probes, and 89% contained more than 10,000 probes, which are unlikely to be targeted arrays. These results demonstrated a robust association between differential expression and disease variants.

Based on the observed associations, we created a tool called fitSNPs to prioritize disease genes from candidate GWAS loci. First, we successfully distinguished true disease genes from false positives (positive SNPs from initial scan subsequently found to be negative during validation) for T1DM GWASs with 89% specificity and 75% sensitivity, and T2DM GWASs with 85% specificity and 60% sensitivity. We then directly rediscovered true T1DM genes by analyzing the top seven loci of WTCCC GWAS initial scan results using fitSNPs. Furthermore, in an unexplained locus (4q27), fitSNPs predicted that a novel gene, *KIAA1109*, may explain the association for T1DM and several autoimmune diseases. We also examined the findings of a segmental copy number variation (CNV) study [49], which was performed using a whole-genome tiling-path bacterial artificial chromosome array to detect a gain or loss of more than 40 kilobases in 93 human samples. The results were uploaded into the UCSC genome browser as a custom track. Using the custom track, we found a CNV in *KIAA1109*, suggesting that CNV might play a role in T1DM.

Although there are existing gene prioritization methods, this is the first to describe the use of differential expression to systematically prioritize candidate genes or SNPs. We acknowledge that no single gene prioritization method is perfect and suggest that fitSNPs can also be used in a complementary manner with other prioritization methods. Given that there are more than 100 published GWASs, we believe that fitSNPs can serve as an effective tool to systematically prioritize candidate SNPs from them.

In theory, FitSNPs can also be used to design SNP arrays for GWASs. It has been shown that tagSNPs could lower costs by 53% while capturing 80% of common SNPs in the African population [50]. In comparison, a DER of 0.48 achieved similar sensitivity; 57% of genes in the genome have a DER value larger than 0.48. They comprise 74% of genes known to have disease-associated variants. A GWAS focusing on these genes could lower experimental costs by 43% while covering at least 74% of disease genes. Therefore, fitSNPs could reduce GWAS costs in a way comparable to that of tagSNPs, but with the additional advantages of gene prioritization and direct linkage to functional experiments. Furthermore, fitSNPs could be combined with tagSNPs in the design of GWASs to further reduce costs and to expedite the discovery of causative genes and DNA variants.

To facilitate the use of fitSNPs, we developed a web server [51] that retrieves DER values, and a comprehensive list of validated and predicted disease associations for all human genes and their underlying microarray study results.

## Conclusion

This study demonstrates that highly differentially expressed genes are more likely to harbor disease-associated variants. FitSNPs successfully distinguished true disease genes from false positives of GWASs for multiple diseases, and can serve as a powerful and convenient tool to prioritize disease genes from GWASs. We further proposed 2,586 genes to sequence for 597 syndromes with unknown molecular basis. With the wealth of genomic, genetic, and disease databases in public international repositories, we are now able to investigate systematically the molecular and genetic mechanisms of diseases, make predictions, and validate them using commercial kits and core facilities. To maximize their value, these molecular measurements must be placed within the context of physiology. A public repository of de-identified clinical measurements will greatly accelerate this process [52].

## Materials and methods

### GEO datasets

The GEO contains gene expression profiles for more than 200,000 individual microarray samples. They are assembled into biologically meaningful and comparable GEO datasets with manually annotated experimental details, such as variables that were studied in the experiment. All samples within a GEO dataset were measured on the same platform with the same background processing and normalization, and their values were directly comparable. We downloaded, processed, and annotated all GEO datasets from GEO, and obtained 476 human GEO datasets, in which both the GEO platform and the GEO dataset were annotated as human.

### Differentially expressed genes

Each GEO dataset was categorized into subsets annotated with one of the 24 types, including disease state, genotype/variation, strain, infection, development stage, age, time, agent, dose, tissue, cell type, cell line, metabolism, stress, growth protocol, protocol, gender, individual, isolate, shock, species, specimen, temperature, and others. We performed all possible subset-versus-subset comparisons in each comparison type in every GEO dataset, ignoring subsets with fewer than three samples. For every comparison, we identified all differentially expressed probes using two class unpaired analysis in the R package of SAM (SAMR) with version 1.25 [28]. We used all default parameters with standard *t*-statistics: nperms = 50, fold > 0, and delta < 0.4. All differentially expressed probes with *q *≤ 0.05 were recorded and annotated with the latest Entrez Gene IDs using AILUN [27]. For 4,552 out of 4,877 comparisons, at least one gene exhibited a significant difference.

### DER

The DER was calculated for each Entrez Gene ID as the count of GEO datasets in which it was differentially expressed divided by the count of GEO datasets in which it was measured. Genes measured in fewer than 5% of GEO datasets were removed.

### Disease genes

Human genes with known disease-associated variants were downloaded from HGMD Professional (Biobase) and GAD. HGMD gene symbols were related to Entrez Gene IDs using AILUN [27]. Entrez Gene IDs were retrieved from GAD entries with validated disease associations, and compared with the latest Entrez Gene ID list to replace or remove outdated Gene IDs and nonhuman genes.

### Differentially expressed genes versus disease genes

For a cutoff from 0 to 1 with an increment of 0.02, differentially expressed genes with DER values greater than the cutoff were compared with the list of disease genes to calculate the precision and recall. Constantly expressed genes with DER less than the cutoff were similarly evaluated. For the control, the label of disease genes was also shuffled 10,000 times within all human genes and compared with differentially expressed genes.

### Comparison of DER values for T1DM genes with those of false positives in GWASs

For each of top seven loci described in the WTCCC T1DM GWAS [31], all genes with described validation results were manually extracted from the paper and supplementary materials [30,31]. The DER values were compared between T1DM and non-T1DM genes in accordance with the validation result.

### Comparison of DER values for T2DM genes with those of false positives in GWASs

T2DM genes were extracted from six T2DM GWASs [29,32-36] and tens of association studies. Of them, genes associated with T2DM in three or more populations were recorded as true T2DM genes. False-positive SNPs were extracted from Table S7 of the report of a T2DM GWAS [32], which were found to be positive in the stage 1 GWAS but found to be unassociated with T2DM during the validation phase, with p value from permutation larger than 0.05. They were annotated with Entrez Gene IDs using Entrez dbSNP [53]. All SNPs without gene annotations were removed.

### FitSNPs

FitSNPs [39] is a list of human Entrez Gene IDs with DER values. Genes with disease-associated variants and corresponding diseases were retrieved from HGMD [26] and GAD [25]. To facilitate integration between fitSNPs and GWASs on the human genome, all reference SNPs were downloaded from dbSNP [53] and assigned DER scores according to their associated genes. For SNPs mapping to multiple genes, the highest DER value was selected. FitSNPs can be loaded into the UCSC genome graph, in accordance with the instructions in the GWAS page of the FitSNPs server [37]. It will automatically show up as a custom track in the UCSC genome browser that can be compared with a wealth of genomic data, including multiple GWAS study results.

### Predicting T1DM genes from the top seven loci of GWASs

Both DER values and WTCCC T1DM GWAS -log_10_*P *were visualized in UCSC genome browser [54] for the top seven loci. Genes with DER value ≥ 0.55 and -log_10_*P *>5 were predicted to be T1DM genes, and compared with the validation findings.

### Mapping diseases without known molecular basis to lead genes

All diseases in OMIM morbid map with a percentage preceding their MIM numbers were considered to be Mendelian disorders without known molecular association. Cytogenetic locations of these diseases and all human genes were retrieved from the OMIM morbid map and the Human Gene Nomenclature Committee, respectively. Highly differentially expressed genes with DER ≥ 0.55 were identified from the cytogenetic location for each disease. Within them, genes that have not been known to have disease-associated variants were predicted to be associated with a corresponding disease.

## Abbreviations

CNV: copy number variation; DER: differential expression ratio; fitSNPs: functionally interpolating single nucleotide polymorphisms; GAD: Genetic Association Database; GEO: Gene Expression Omnibus; GWAS: genome-wide association study; HGMD: Human Gene Mutation Database; OMIM: Online Mendelian Inheritance in Man; SAM: significance analysis of microarrays; SLE: systemic lupus erythemetosus; SNP: single nucleotide polymorphism; T1DM: type 1 diabetes mellitus; T2DM: type 2 diabetes mellitus; UCSC: University of California Santa Cruz; WTCCC: Wellcome Trust Case Control Consortium.

## Authors' contributions

RC designed and performed the experiments, and wrote the manuscript. AB provided the overall project guidance. AC provided critical review and edited the manuscript. KK collected T2DM gene lists and gave advice on the validation. AM, JD, TD, and LL gave advice on the experiments. All authors read and approved the manuscript.

## Additional data files

The following additional data are available with the online version of this paper. Additional data file 1 is an enrichment comparison between genes differentially expressed in one or more microarray studies and genes with disease-associated variants.

## Supplementary Material

Additional data file 1 Presented is an enrichment comparison between genes differentially expressed in one or more microarray studies and genes with disease-associated variants. Click here for file
