# Supplementary material for: FitSNPs: highly differentially expressed genes are more likely to have variants associated with disease
Source: Genome Biol. 2008 Dec 5;9(12):R170. doi: 10.1186/gb-2008-9-12-r170 (PMC2646274; doi:10.1186/gb-2008-9-12-r170)
Supplement: Additional data file 1 — Presented is an enrichment comparison between genes differentially expressed in one or more microarray studies and genes with disease-associated variants. [file gb-2008-9-12-r170-S1.pdf]

Additional data file 1

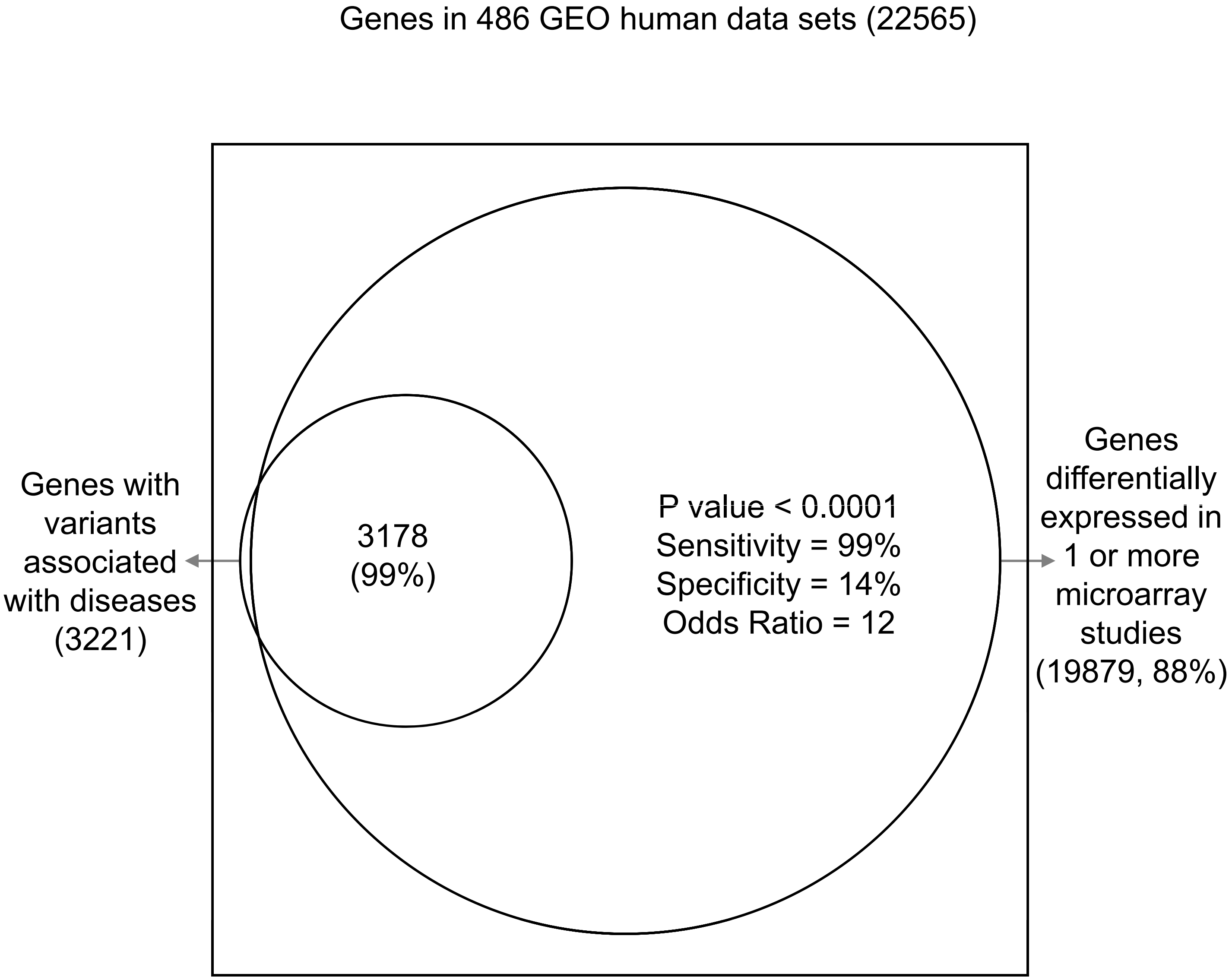

Additional data file 1: Comparison between genes differentially expressed in one or more microarray studies and genes with disease-associated variants. Genes that were differentially expressed in one or more microarray studies were identified using SAM with q value ≤ 0.05. These genes were used to rediscover genes with disease-associated DNA variants listed in GAD and HGMD. The process allowed us to calculate the sensitivity and specificity. The odds ratio was calculated as the probability ratio between differentially and constantly expressed genes to rediscover disease genes. Constantly expressed genes were not differentially expressed in any microarray studies. P value was calculated using Fisher's exact test.
